# Suture Technique to Prevent Air Leakage during Negative-Pressure Wound Therapy in Fournier Gangrene

**DOI:** 10.1097/GOX.0000000000001650

**Published:** 2018-01-25

**Authors:** Feng-Shu Chang, Chieh Chou, Chuan-Yu Hu, Shu-Hung Huang

**Affiliations:** From the * Division of Plastic Surgery, Department of Surgery, Kaohsiung Medical University Hospital, Kaohsiung Medical University, Kaohsiung, Taiwan; † Department of Nursing, Kaohsiung Medical University Hospital, Kaohsiung, Taiwan; ‡ Department of Surgery, Faculty of Medicine, Collage of Medicine, Kaohsiung Medical University, Kaohsiung, Taiwan; and § Center for Stem Cell Research, Kaohsiung Medical University, Kaohsiung, Taiwan.

## Abstract

**Background:**

The use of negative-pressure wound therapy (NPWT) for Fournier gangrene management is well documented; however, it is difficult to fixate GranuFoam dressings and maintain an airtight seal over the perineum area. We developed a simple method to facilitate GranuFoam fixation and improve airtight sealing.

**Methods:**

The Fournier’s gangrene severity index (FGSI) score less than 9 was collected in from January 2015 to October 2016. All 13 patients underwent fasciotomy, and NPWT was applied directly on fasciotomy wounds after the debridement of infected tissue. Partial wound closure was performed, and a portion of GranuFoam was inserted to facilitate fixation. The seal check was converted to a 0–10 scale score that was recorded every 4 hours during NPWT. Patient profiles including medical history, FGSI, method of wound closure, and length of stay were collected in this study.

**Results:**

The median age of the patients was 62 (38–76) years. The mean FGSI score was 4.3 ± 3.1. The average duration of NPWT was 17.5 ± 11.5 days, and the average seal check score was 0.8 ± 0.5. No seal check alarms were noted during the study. Successful wound closure was achieved in all patients without using additional reconstruction methods such as skin grafting or muscle flap coverage.

**Conclusions:**

The present results suggest that partial wound-edge closure and in situ GranuFoam fixation improve the NPWT leaks in Fournier gangrene wounds. Furthermore, this method is simple to learn and can be useful in applying NPWT to anatomically difficult areas.

## INTRODUCTION

Fournier gangrene is a surgical emergency disease with a mortality rate of over 40%; it is characterized as an infectious necrotizing fasciitis, mostly caused by skin infections and affects the external genitalia, perineum, or perianal regions.^1–4^ This soft-tissue infection involves polymicrobial organisms such as *Escherichia coli*, *Streptococcus pyogenes*, *Pseudomonas aeruginosa*, *Klebsiella pneumoniae*, *Enterococci* spp, *Bacteroides fragilis*, and anaerobic streptococcus that cause an ultimate thrombosis of the subcutaneous vessel and lead to skin necrosis.^3–5^ Current treatments include intensive surgical debridement, systemic antibiotic administration, stool diversion (if necessary), and wound bed preparation using biological dressings or negative-pressure wound therapy (NPWT), followed by reconstructive procedures.^5–9^

After initial surgical debridement, several subsequent operations are required. During wound bed preparation, a high level of wound exudate discharge is commonly observed in affected patients; therefore, an effective dressing is crucial for the control of the overload in the exudative discharge and the removal of bacterial load.^10^ NPWT has become the mainstay in the management of Fournier gangrene.^3,10^ NPWT facilitates the wound healing process physiologically by reducing edema, removing infectious materials and exudates, and increasing blood supply. Furthermore, the negative pressure accelerates the formation of granulation tissues compared with traditional dressing methods.^7,11,12^ Arslan et al.^13^ demonstrated the positive effects of NPWT on plasma fibronectin levels and revealed that fibronectin promotes the migration of inflammatory cells and improves wound healing. In addition to its well-documented physiological effects, NPWT has other advantages such as requiring less frequent changes in wound dressing, less pain, fewer skipped meals, and greater mobility compared with conventional dressing methods.^6^

The use of NPWT for the management of Fournier gangrene has been well documented^3,5,7^; however, the complex contours of the perineum areas present a major challenge in the sealing of the negative-pressure dressing. Moreover, it is difficult to place the foam dressing over irregular surfaces surrounding the wound and maintain an airtight seal. Therefore, the present report describes the outcomes of the treatment of a series of complex Fournier gangrene wounds by using a novel technique to achieve an effective air sealing of the NPWT dressing.

## METHODS

From January 2015 to October 2016, we examined patients with Fournier gangrene having a Fournier’s gangrene severity index (FGSI) score of less than 9.^4^ Each patient underwent fasciotomy and debridement of infected tissue followed by NPWT (V.A.C. Therapy, KCI [Acelity Company], San Antonio, Tex.). A foam dressing was placed in the entire wound cavity. Where possible, the wound sections were primarily sutured together over the foam to allow skin flap preservation. The areas of closed-wound edges created pockets to help hold the foam in place and maintain foam contact with the wound bed. The drape was sealed over the wound and periwound skin, and continuous NPWT was applied at −125 mm Hg. Dressing changes were performed 2 times per week under general anesthesia according clinical condition. The seal check bar height was recorded whenever this suture technique is performed. Empirical antibiotics were administered upon admission. After culture results were obtained, each patient was administered culture-specific antibiotics.

**Fig. 1.**
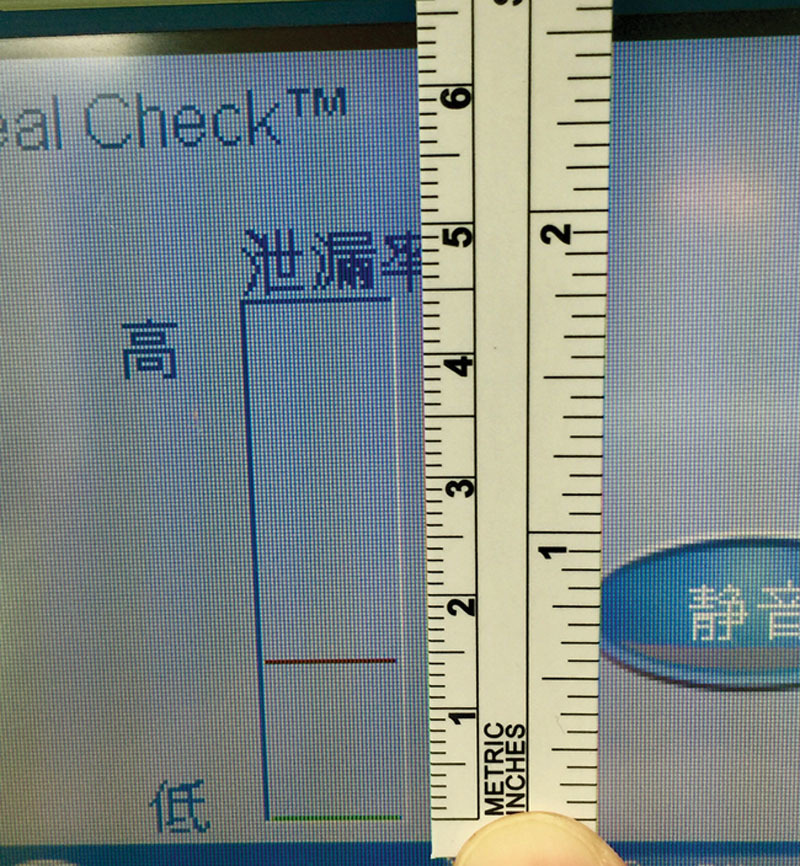
Seal check on the NPWT device measured by a ruler. The total bar length is 45 mm and was converted to a 0–10 scale score.

In the present case series, we converted the seal check indicator on the NPWT device to a 0–10 scale score to quantify dressing leaks after NPWT (Fig. 1). The total length of the seal check indicator bar for the NPWT device was 45 mm. We used the following equation to convert the height of the seal check bar into a quantitative measure:


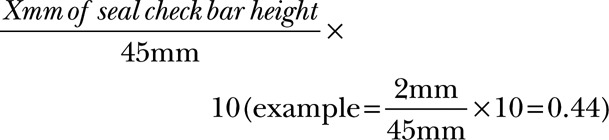


Therefore, a lower score represents fewer dressing leaks. The seal check score and the frequency of the seal check alarm were recorded every 4 hours during NPWT, and the means of the scores were also recorded. In addition, the frequency of the seal check alarm was recorded. Parameters such as the body mass index, number of operation (ie, debridement, fasciotomy, fasciectomy, and closure of the wound were the operations we accounted for), method of wound closure, and length of stay (LOS) were also examined in this study.

## RESULTS

From January 2015 to October 2016, 13 patients were treated, of whom 11 were male and 2 were female (Table 1). The median age of the patients was 62.5 years (56–73 years), and the mean FGSI score was 4.3 ± 3.1. On average, the patients received 17.5 ± 11.5 days of NPWT, and the average LOS was 26.5 days. With our novel NPWT dressing application technique, a reliable airtight seal was achieved in all patients. The average seal check score was 0.27 ± 0.14, and no seal check alarms were noted during the study. All patients survived, and successful wound closure was achieved in all patients without the requirement of other reconstruction methods such as skin grafting or muscle flap coverage.

**Table 1.**
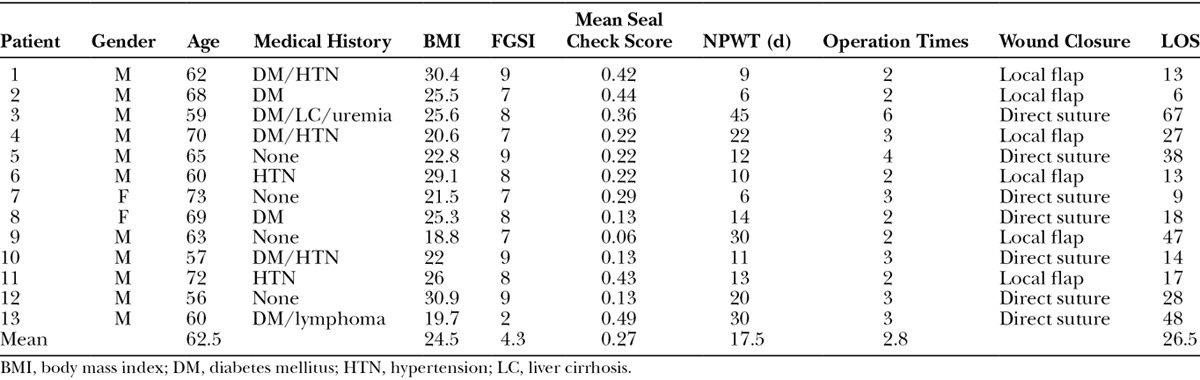
Patient Demographics and Outcomes

### Representative Cases

**Fig. 2.**
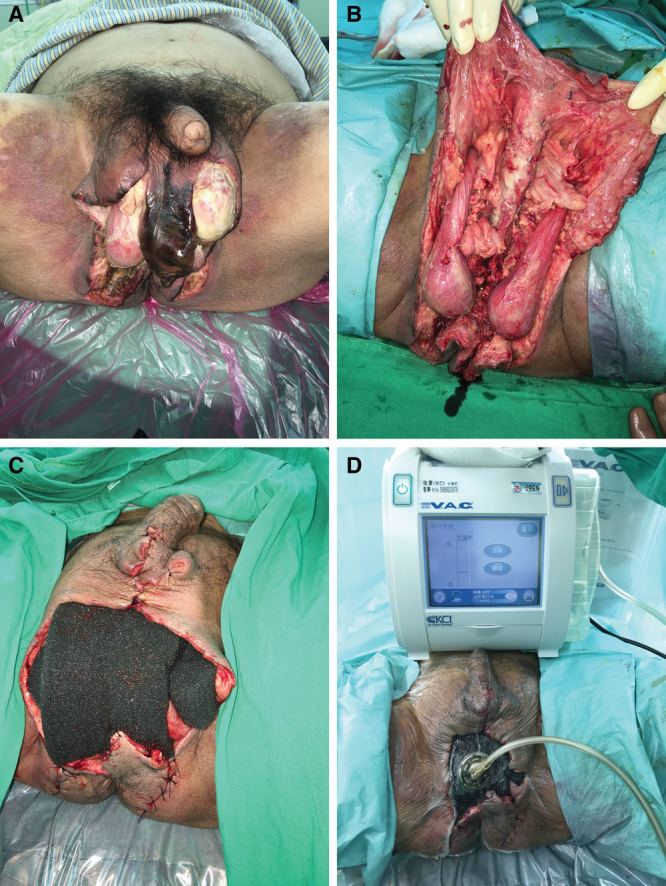
A 59-year-old man with underlying diabetes mellitus, liver cirrhosis, and uremia developed Fournier gangrene extending to the scrotum, perianal area, and pubic area. A, Wound area before second operation. B, Postoperative wound with testis exposure and extending to the perianal area. C, Partial wound closure by the insertion of the NPWT-reticulated open-cell foam dressing. D, NPWT applied to the wound without leakage.

A 59-year-old man (case 3) with underlying diabetes mellitus, liver cirrhosis, and uremia developed Fournier gangrene extending to the scrotum, perianal area, and pubic area (Fig. 2A). The patient’s FGSI score was 8. Extensive surgical debridement was performed, leading to the exposure of the testes and extension of the wound to the perianal area (Fig. 2B). NPWT was applied with a simple wound-edge closure as described previously (Fig. 2C). No air leaks were detected after NPWT (Fig. 2D). Due to infection progress, the patient had undergone operation 6 times, and the wound was closed with staged closure. The LOS was 67 days.

**Fig. 3.**
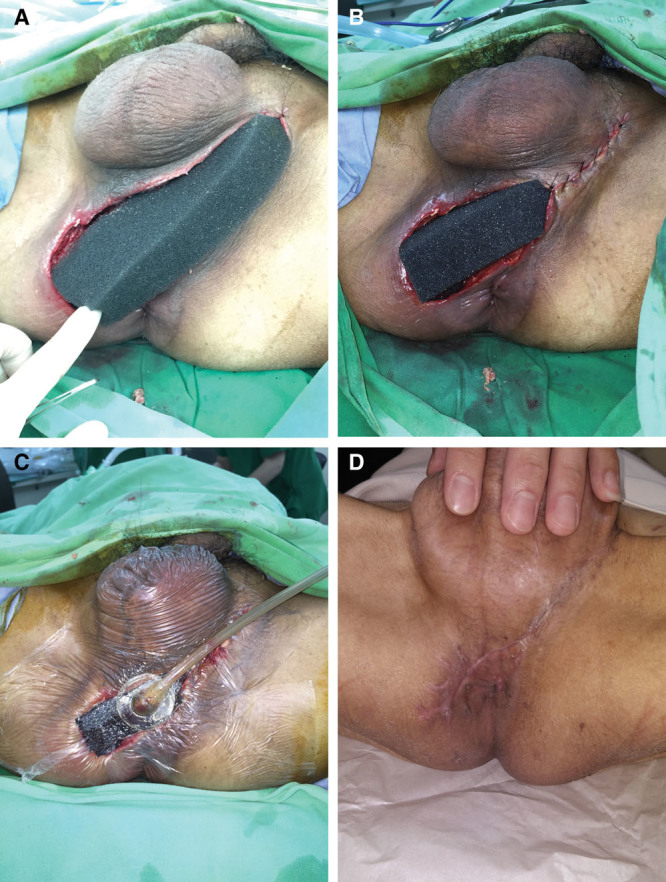
A 63-year-old man with diabetes mellitus and lymphoma developed Fournier gangrene. A, Trimmed GranuFoam placed into the wound postfasciotomy. B, Skin around the wound was partially sutured to secure the foam into the wound cavity. C, NPWT applied to the perineum area. D, Healed wound following delayed closure.

A 60-year-old man (case 13) with diabetes mellitus and lymphoma developed Fournier gangrene and presented with an FGSI score of 7 (Fig. 3). Part of the wound edge was sutured together to secure the NPWT foam (Fig. 3A, B). NPWT was applied easily on the perineum area (Fig. 3C). The wound healed following delayed closure (Fig. 3D). He underwent operation 3 times and had a mean seal check score of 0.49 during NPWT.

## DISCUSSION

All 13 patients in the present case series survived and underwent successful wound closure within an average LOS of 26.5 days with the use of NPWT and the novel foam dressing application technique. Despite the difficult wound anatomy, this technique enabled achieving a reliable negative-pressure airtight seal over all wounds and maintaining it throughout the therapy period. A seal check score of ≤ 1 was obtained at all time points in all patients, indicating the presence of minimal air leaks.

Upon admission, the patients were assessed for disease severity based on their FGSI scores. Laor et al.^14^ developed the FGSI score to determine the severity of Fournier gangrene. The FGSI score, developed to determine disease severity and predict patient prognosis, is obtained from a combination of hospital admission parameters (temperature; heart rate; respiration rate; serum sodium, potassium, and creatinine; hematocrit; white blood count; and serum bicarbonate). Tarchouli et al.^15^ assessed 72 patients with Fournier gangrene and observed that the median FGSI score was significantly higher in nonsurvivors (*P* = 0.002). An FGSI score of 9 was used as the threshold parameter for outcome prediction. FGSI scores of ≥ 9 and < 9 had a 75% and 78% probability of death and survival, respectively.^14,15^ Therefore, the FGSI is a simple and effective tool for predicting Fournier gangrene severity and patient survival. Recently, Yilmazlar et al.^16^ suggested a new scoring system, namely the Uludag FGSI, involving the addition of a physiological score (the age and extent of gangrene) to the traditional FGSI score. In our patients, the FGSI scores varied between 0 and 10, and 3 of the 13 patients had an FGSI score of > 7, indicating high severity scores. Although the 0% mortality rate in our patients is encouraging, studies on a considerably larger population size are warranted.

Maintaining an adequate airtight seal is critical in providing optimal NPWT dressing. Seal leaks can cause pain and discomfort to the patient, waste valuable nursing time, engender desiccation, and delay wound healing.^17^ A previous study examined the clinical effects of air leaks in a porcine wound model.^18^ Four porcine wounds were treated with NPWT, and an unregulated air leak in the sealing drape resulted in a significant progression of wound dehydration and necrosis (*P* < 0.0001). This finding reconfirms that air leaks in clinical wounds result in wound deterioration and prolong the wound healing time and increase the number of surgical procedures. The described study also revealed a negative pressure of −125 mm Hg to be the optimal pressure for granulation formation.^18^ In our case series, the pressure was set at −125 mm Hg to yield optimal results. The leak detection feature on NPWT devices can alert the health care teams to check dressing leaks, which can be eventually used to provide an optimal therapy. The ultimate goal of this technique is the complete elimination of air leaks and leak alarms to reduce patient and clinician anxiety and enable the effective continuation of NPWT.^19^ All our patients had a seal check score of ≤ 1 at all time points during NPWT, indicating the presence of minimal to no air leaks.

In patients with Fournier gangrene with perianal involvement, fecal contamination is a common complication; therefore, fecal diversion should be considered in the treatment of Fournier gangrene. Colostomy is the most common procedure for fecal diversion; however, it requires subsequent colostomy closure after months.^20^ Recently, the Flexi-Seal Fecal Management System (ConvaTec, Inc., Skillman, N.J.) has been reported to be suitable for short-term fecal diversion to prevent the fecal contamination of perineum wounds; this system is an alternative treatment choice to colostomy and avoids additional operation or colostomy complications.^21–23^ Eleven of our patients underwent colostomy due to the severity of Fournier gangrene and extensive perineum involvement.

The use of NPWT for the management of Fournier gangrene wounds has become a gold standard. NPWT is an effective method to clean and prepare wounds for closure compared with conventional dressing techniques. This method facilitates removing exudates, blocking inflammatory process, reducing the frequency of wound dressing changes, lessening the pain, reducing the number of skipped meals, providing greater mobility, and reducing the LOS.^5–7^ Our results are consistent with the positive outcomes of the aforementioned studies. However, this method has some disadvantages and limitations such as general anesthesia is needed for change NPWT, inadequate debridement, and huge skin defects (skin loss in the perineal area after debridement procedure). Additionally, skin margin necrosis due to excess tension in suture traction might be a complication.

The rapid and aggressive pathological processes in Fournier gangrene can cause scrotal skin, perineum, and abdominal defects in severe cases. The principle of surgical reconstruction is based on the characteristics of defects, namely the size, location, and depth, as well as the availability of local tissues in the scrotum or thigh area. Karian et al.^24^ devised an algorithm for the reconstruction of Fournier gangrene defects. If the defect is < 50% of the scrotum, delayed primary closure is suggested when there is no tension. Under the presence of tension, the scrotum defects are closed with local scrotal advancement flaps or wound healing is provided through secondary intervention. If the defect involves > 50% of the scrotum or extends beyond the scrotum, split-thickness skin graft or flap reconstruction is suggested.^24^ The ideal reconstructive technique would be a single procedure, yielding optimal function and aesthetic appearance of the wound with minimal postoperative and donor-site complications. In the present case series, most patients underwent delayed primary closure or local scrotal advancement flap procedure, because the defect size was reduced to small and medium after NPWT.

## CONCLUSIONS

Our results suggest that partial wound edge-closure for foam fixation reduces air leaks during Fournier gangrene wound dressing using NPWT. This method is simple to learn and is useful, particularly for anatomically difficult areas.

## ACKNOWLEDGMENTS

The authors thank Julissa Ramos, PhD, and Julie M. Robertson, PhD, for their assistance in the article preparation and editing. The authors gratefully acknowledge Miss Yen-Hsin Kuo for providing technical assistance and preparing the figures in this article.
